# Clear Skies and Grey Areas: Flight Attendants’ Secondhand Smoke Exposure and Attitudes toward Smoke-Free Policy 25 Years since Smoking was Banned on Airplanes

**DOI:** 10.3390/ijerph120606378

**Published:** 2015-06-04

**Authors:** Frances A. Stillman, Andrea Soong, Laura Y. Zheng, Ana Navas-Acien

**Affiliations:** 1 Department of Health, Behavior and Society, Johns Hopkins Bloomberg School of Public Health, Baltimore, MD 21205, USA; E-Mail: asoong@jhu.edu; 2 Department of Environmental Health Sciences, Baltimore, Johns Hopkins Bloomberg School of Public Health, MD 21205, USA; E-Mails: lzheng15@jhu.edu (L.Y.Z.); anavasa1@jhu.edu (A.N.-A.)

**Keywords:** secondhand smoke, environmental health, occupational health, public health policy, flight attendants, travel

## Abstract

Our objective was to provide descriptive data on flight attendant secondhand smoke (SHS) exposure in the work environment, and to examine attitudes toward SHS exposure, personal health, and smoke-free policy in the workplace and public places. Flight attendants completed a web-based survey of self-reported SHS exposure and air quality in the work environment. We assessed the frequency and duration of SHS exposure in distinct areas of the workplace, attitudes toward SHS exposure and its health effects, and attitudes toward smoke-free policy in the workplace as well as general public places. A total of 723 flight attendants participated in the survey, and 591 responded to all survey questions. The mean level of exposure per flight attendant over the past month was 249 min. The majority of participants reported being exposed to SHS always/often in outdoor areas of an airport (57.7%). Participants who worked before the in-flight smoking ban (n = 240) were more likely to support further smoking policies in airports compared to participants who were employed after the ban (n = 346) (76.7% *versus* 60.4%, *p*-value < 0.01). Flight attendants are still being exposed to SHS in the workplace, sometimes at concerning levels during the non-flight portions of their travel. Flight attendants favor smoke-free policies and want to see further restrictions in airports and public places.

## 1. Introduction

Twenty-five years ago in February of 1990, smoking was banned on all U.S. domestic short-haul flights [1]. This was followed by a ban on smoking on all U.S.-based international flights in 2000 after a decades-long push for a policy to eliminate smoking on all aircrafts and repeated tobacco industry efforts to interfere [1,2,3]. Existing research on flight attendants has evaluated the health status of those employed before and after the milestone ban, with a focus on respiratory health [4,5], long and short-term health effects [6], cause-specific mortality [7], and comparisons of health status with the general population [8]. Secondhand smoke (SHS) exposure is an occupational hazard for flight attendants, regardless of personal smoking habits or whether they worked on planes when smoking was allowed. Repace’s review of the literature on SHS and air pollution in aircraft cabins found that regardless of whether flight attendants worked in a smoking or nonsmoking plane section in the past, SHS exposure was higher than the general population [9]. Designated smoking rooms (DSRs) in airports remain common, even in countries that have bans on smoking in most or all public places [10]. This is despite repeated evidence that they do not effectively protect outside air from SHS contamination [11,12,13,14,15].

This paper focuses on exposure to secondhand smoke among flight attendants in places frequented during international work travel, including but not limited to airports, hotels, and restaurants. Specifically, the aim of the study was to describe flight attendant SHS exposure in the workplace, including where they are exposed to during working shifts (airports, non-air transportation, restaurants, and hotels) and how much. We also evaluated flight attendant attitudes toward SHS exposure and its relation to their personal health, as well as their attitudes toward smoke-free workplace and public place policies. These results can be used to shape future policy and education efforts to reduce SHS exposure among flight attendants as well as air travelers.

## 2. Methods

### 2.1. Study Population

A web-based survey of flight attendants, SHS exposure and air quality in the work environment was conducted during the summer of 2012. Institutional Review Board (IRB) approval was obtained from the Johns Hopkins Bloomberg School of Public Health, and participants gave written consent for participation in the study prior to starting the survey. Participants were recruited through an electronic flyer containing a description of the study and link to the survey that we provided to a major flight attendant union, which distributed the flyer by email to their members. The email with the flyer was only sent once and there were no reminders to participate. In addition, we posted online advertisements on Facebook containing a brief description and link to the survey. These ads were targeted to users who were subscribed to airline or flight attendant groups or pages, were based in the U.S., and were between the ages of 18 and 65. The ad reached a total of 93,630 users.

Formative discussions with flight attendants and key informants determined eligibility criteria for the study. Eligible participants included attendants who worked at least one year in their current position and serviced a minimum of three international flights per month. Other work metrics included which airline they worked for, their home airport, the number of days worked in an average month, the frequency and length of international work trips. The study focused on U.S. airline carriers, but the option to participate was open to all English-speaking adult flight attendants. At the start of the survey we asked participants: (1) were they an active flight attendant; (2) had they worked as flight attendant for the past 12 months; (3) did they travel internationally for work; and (4) how many times per month did they travel internationally for work. Participants who answered to any of these questions with an ineligible response were redirected to a disqualification page.

### 2.2. Data Collection

The study questionnaire was adapted from a questionnaire developed for a study of secondhand smoke exposure among bar and nightclub employees, which was conducted in more than 25 countries around the world [16]. Participants self-reported their exposure to SHS over the past month in five areas of airports over the past month: inside restricted/employee only areas, indoor eating areas, outdoor areas (defined as passenger drop-off and pick-up areas, taxi/bus waiting areas), indoor public areas, and near or outside designated smoking rooms. Participants were asked to self-report how frequently they were exposed to SHS in these various areas: always/often, sometimes/rarely, and never. Next, they were asked to enter the duration of SHS exposure in minutes for these same airport areas. These questions on frequency and duration of SHS exposure were repeated for nine different public places visited during international work-related travel in the past month, including indoor and outdoor areas of hotels, restaurants, cafes, and bars. Validity of the secondhand smoke exposure questions had been previously validated against hair nicotine concentrations, a biomarker that reflects several months of exposure to secondhand smoke [16].

The survey asked about participants’ attitudes toward SHS exposure and its health effects (“Do you feel that your health has been compromised by occupational exposure to secondhand smoke?”). The survey asked about attitudes toward smoke-free work places and policies (“Do you prefer to work in a smoke-free environment?” and “Do you think that indoor and/or outdoor public places should be smoke-free?” and “Do you believe that airports need to implement further policies to control tobacco smoking within or outside the airport?”). Other questions in the survey focused on participants’ health issues including the presence and severity of respiratory symptoms, and these results were studied in-depth by Shargorodsky *et al.* [17].

### 2.3. Measures

The percentage of participants who responded to SHS exposure in various areas was calculated. Numerical variables were generated from the number of minutes of reported exposure to SHS in each of the areas of airports and public places. The number of minutes from each location was summed to create a variable for total SHS exposure (in minutes) and this variable was then divided into three tertiles.

### 2.4. Statistical Analysis

A total of 723 flight attendants participated in the study. Descriptive analyses were performed for demographic characteristics and SHS exposure variables. Chi Square was used to compare across groups. For this study, we excluded 132 participants who had missing values for age, gender, and country of origin, leaving 591 flight attendants. Continuous variables were grouped into tertiles according to SHS exposure in minutes. For prevalence ratios, logistic regression was used to calculate marginal prevalences and prevalence ratios. The delta method was used to calculate 95% confidence intervals [18]. All analyses were performed with Stata 13.0 (Stata Corporation).

## 3. Results

Table 1 lists the sample characteristics. Female participants comprised most of the sample (68.9%). More than two-thirds of the sample (67.0%) reported that they had never smoked. The majority of participants (87.0%) worked for a US carrier, and serviced an average of 5.2 international trips per month. The average number of years worked as a flight attendant was 16.1.

**Table 1:** Participant characteristics.

**Characteristic**	Overall	Pre-Smoking Ban	Post-Smoking Ban	*p*-Value
**N**	591	346	242	
**Gender**				
**Male**	31.1	24.0	64.2	<0.01
**Female**	68.9	76.0	35.8	
**Age**	42.9 (11.7)	52.3 (7.0)	36.4 (9.7)	<0.01
**Country of origin**				
**US**	87.0	92.6	83.0	<0.01
**Non-US**	13.0	7.4	17.0	
**Smoking Status (N = 521)**				
**Never**	67.0	61.0	71.4	<0.01
**Former**	19.3	25.7	14.7	
**Current**	13.7	13.3	13.9	
**Airline Carrier (N = 571)**				
**US Carrier**	96.2	99.6	93.6	<0.01
**Non-US Carrier**	3.8	0.4	6.4	
**Years worked as flight attendant (N = 586)**	16.1 (11.1)	27.1 (7.3)	8.4 (5.4)	<0.01
**Number of international trips/month**	5.2 (3.7)	4.6 (2.7)	5.7 (4.3)	<0.01
**(N = 582) Number of hours per international trip**	9.3 (5.1)	9.5 (4.4)	9.0 (5.1)	0.17
Current SHS exposure, min * (N = 528) ≤ 45	33.5	41.0	28.4	<0.01
**48–147**	34.1	32.6	35.1	
≥**148**	32.4	26.4	36.4	

Data in the table are mean (SD) or percentages. *p*-values calculated by t-test or χ^2^ test. * The sample size for current SHS was 528, 212, and 313 for the overall, pre-smoking ban and post-smoking ban samples, respectively.

### 3.1. Exposure

The mean level of exposure per flight attendant over the past month was 249 min (range 8 to 440 min at the 10th and 90th percentile, respectively). Reports of current SHS exposure by participants who worked pre- and post-smoking ban (41.0% and 28.4%, *p*-value < 0.01) differed significantly.

**Figure 1:**
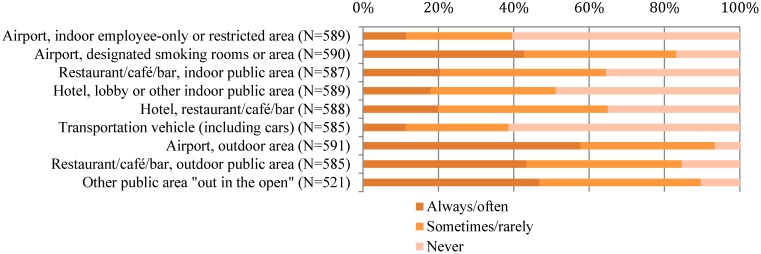
Flight attendant areas of secondhand smoke exposure and frequency of exposure.

Figure 1 displays the proportion of participants who reported being exposed to SHS in various airport areas and venues, by the frequency at which they were exposed (always/often, sometimes/rarely, never). The majority of participants reported being exposed to SHS always/often in outdoor areas of an airport (57.7%) (Figure 1). Less than half reported always/often SHS exposure near designated smoking rooms or areas in airports (42.7 %), approximately half reported sometimes/rarely exposure in hotel restaurants (45.1%), and half reported never being exposed in hotel lobbies or indoor public areas (48.9%).

### 3.2. Attitudes

Table 2 lists participant attitudes toward smoke-free policy and personal health. In response to the question “Do you believe that airports need to implement further policies to control tobacco smoking within or outside airports?” 76.7% of participants who worked before the in-flight smoking ban (n = 240) responded “yes,” *versus* 60.4% of participants who were employed after the ban (n = 346).

Table 3 contains prevalence ratios of responses to the same question (“Do you believe that airports need to implement further policies to control tobacco smoking within or outside the airport?”) before and after adjustment for age, gender, smoking status, pre/post ban work status, and self-reported information on been affected by secondhand smoke exposure.

After adjustment for other characteristics, current smokers were less likely to support implementing further smoke-free policies in airports (prevalence ratio 0.44; 95% CI 0.31, 0.36). Participants who indicated that they had been affected by SHS exposure were more likely to support further smoke-free policies in airports (prevalence ratio 1.71; 95% CI 1.46, 2.01). The association of support of further policies with age and with working when smoking was allowed in the planes was markedly attenuated after adjustment for other characteristics, with the prevalence ratio (95% CI) changing from 1.17 (1.03, 1.35) to 0.96 (0.82, 1.12) for participants ≥50 *vs*. ≤36 years of age and from 1.19 (1.08, 1.32) to 1.08 (0.96, 1.21). This attenuation was mostly explained by adjustment by working when smoking was allowed and by age, respectively, and could be related to collinearity between age and working when smoking was allowed.

**Table 2:** Participant attitudes toward smoke-free policy and personal health.

Survey Question	Overall Yes Response (%)	Active Pre-Ban (%)	Active Post-Ban (%)	*p*-Value *
Do you believe that airports need to implement further policies to control tobacco smoking within or outside the airport? (N = 589)	66.9	76.7	60.4	<0.01
Do you think that indoor public places (airports, restaurants, hotels) should be smoke free? (N = 584)	87.3	92.9	86.6	<0.01
Do you prefer to work in a smoke free environment? (N = 587)	91.5	96.7	87.8	<0.01
Do you think that outdoor public places (outdoor waiting areas, patios, terraces in airports, restaurants, hotels) should be smoke free? (N = 582)	69.6	76.4	65.2	<0.01
Do you feel that your health has been compromised by occupational exposure to secondhand smoke? (N = 588)	59.4	75.9	48.3	<0.01
Do you believe that airports need to implement further policies to control tobacco smoking within or outside the airport? (N = 589)	66.9	76.7	60.4	<0.01

**Table 3:** Prevalence ratios of participant attitudes toward implementing further smoke free policies in airports.

Characteristic	N	Crude PR	Adjusted PR
Age, years			
≤36	173	1.00 (ref)	1.00 (ref)
37–49	194	1.09 (0.95, 1.25)	1.06 (0.95, 1.19)
≥50	179	1.17 (1.03, 1.35)	0.96 (0.82, 1.12)
Gender Male	174	1.00 (ref)	1.00 (ref)
Female	372	0.88 (0.78, 0.99)	1.04 (0.94, 1.14)
Smoking Status Never	363	1.00 (ref)	1.00 (ref)
Former	109	0.95 (0.85, 1.07)	0.96 (0.85, 1.09)
Current	73	0.37 (0.26, 0.53)	0.44 (0.31, 0.63)
Worked when smoking was allowed in planes			
No	313	1.00 (ref)	1.00 (ref)
Yes	230	1.19 (1.08, 1.32)	1.08 (0.96, 1.21)
Current SHS exposure, min			
≤45	156	1.00 (ref)	1.00 (ref)
48–147	165	0.80 (0.68, 0.93)	0.86 (0.76, 0.97)
≥148	164	1.04 (0.92, 1.17)	0.99 (0.88, 1.10)
Reported to be affected by SHS exposure			
No	211	1.00 (ref)	1.00 (ref)
Yes	332	1.84 (1.59, 2.14)	1.71 (1.46, 2.01)

Adjusted PR is adjusted for age, gender, smoking status, pre/post ban work status, SHS exposed, and affected by SHS exposure.

## 4. Discussion 

Flight attendants are still reporting exposure to SHS during international work travel. While no longer exposed during flight, they are still being exposed at differing amounts during their work-related layovers. The average SHS exposure over a month of work was more than four hours (249 min), which is concerning, as only 30 min of exposure can have adverse health effects [19]. The most common area where participants experienced SHS always/often was outdoor areas of airports. This finding implies that further restrictions need to be made on airport smoking policy, for example increasing the distance from entrances where smoking is permitted. Furthermore, there is support in the literature for banning smoking on entire campuses of institutions: A study of outdoor tobacco smoke exposure at a tobacco-free campus measured SHS exposure levels around the smoke-free perimeter and found high levels of PM_2.5_ when standing or passing by boundaries where smokers gathered [20]. Tobacco use is already banned in outdoor and quasi-outdoor areas for the majority of major U.S. public transit systems, thus it is reasonable to apply this policy to airports as well [21].

Indoor SHS exposure was reportedly low, which was expected, as most major airports are either entirely smoke-free, or contain enclosed, separately ventilated smoking rooms (DSR’s). However, these rooms do not effectively prevent SHS leakage [11,12,13,14,15], and flight attendants spend an increased amount of time in airports compared to the general population. Participants reported moderate SHS exposure in areas near these smoking rooms, and similar to previous research these findings show that the allowance of DSR’s remains an occupational health concern [10,11]. It is therefore important that airports implement completely smoke-free indoor policies and eliminate DSRs as it has been done in airports such as Madrid Barajas and Moscow Sheremetyevo [10,22].

Our findings indicate that flight attendants favor smoke-free policies and want to see further restrictions. It is worth noting that support for implementing further smoke-free policies differed significantly by whether participants had worked before (78.2%) or after (61.4%) the U.S. in-flight smoking ban. There is a need to focus SHS exposure and air quality awareness and education efforts toward newer flight attendants, since post-ban participants were less supportive. The long struggle that flight attendants waged to have a smoke-free and healthy workplace may not be as well known to flight attendants employed since the ban was implemented [1,2,3,23]. Not personally experiencing the physical effects and the conditions endured by older flight attendants may explain differences in support for further restrictions on SHS based on the number of years worked as a flight attendant. It is possible that newer flight attendants may not fully understand the benefits gained from these restrictions since they would have only worked smoke-free flights in their career and would not have endured smoke-filled cabins. Research is also needed to ask about support for specific types of restrictions, such as banning smoking in outdoor areas near entrances to airports or in patios as well as attitudes toward removing DSR’s at airports.

The limitations of our study should be noted. This was a convenience sample of flight attendants who self-reported their secondhand smoke exposure and respiratory health history, which may be prone to recall bias. Participants were recruited through a major flight attendant union who agreed to send out a notification of the research study and the link to the online survey to their membership, as well as through a Facebook ad targeted to users who were more likely to be flight attendants. While these methods allowed us to obtain, in a rapid fashion, a sufficient number of respondents who were flight attendants and met the study criteria, we are unable to calculate a response rate without knowing the true denominator. Thus our results should be viewed as possibly biased. They may not be generalizable to a larger sample beyond our study; however, our sample demographics are comparable to other studies of U.S. flight attendants and some similarities and differences should be noted. The participants in our survey were slightly younger than other studies that recruited both male and female participants, with a mean age of 42.9 years, compared to 54 and 47 in Ebbert *et al.* and Mcneely *et al*., respectively [5,8]. There were more male participants in our study—females comprised 68.9% of the sample compared to 80%−89% in similar studies [5,7,8]. The proportion of current smoking was 13.7% in our study, compared to 9% in McNeely *et al*. [8]; ever-smoking (former and current) in our study was 33%, the same as in Beatty, Haight and Redberg (2011) [4]. It is important to consider that while these comparisons are helpful for establishing an idea of the demographic profile of FAs, each study had distinct recruitment criteria and research objectives so this should not be taken out of context.

These study results provide a snapshot into the attitudes, behaviors and current SHS exposure of a large number of flight attendants who travel internationally as part of their work. The methods employed allowed us to tap into a hard-to-reach population to obtain information about their personal situation but can also provide insight into what other flight attendants and travelers might be experiencing during their long-haul travels. The large number of participants allowed for adjustment for multiple potential confounders, thereby strengthening the conclusions.

## 5. Conclusions

Although flight attendants represent a special population and travel constantly, regular travelers too are potentially exposed to SHS in these same airports and areas. Improved airport restrictions on smoking are necessary in order to reduce exposure to SHS in the U.S. and around the world. Increasing smoke-free policies in airports is not likely to be a deterrent to visitors, according to a recent survey of tourists in an international airport in Thailand [24]. The process of banning smoking in commercial airplanes was not simple and took nearly 40 years to be successfully implemented [1,2,3]. However, as more jurisdictions strengthen smoke-free laws in public places, it is reasonable that airports are a natural extension of this in order to protect travelers, patrons, and employees, including the elimination of designated smoking rooms and the banning of smoking in outdoor areas near the main entrances.

## References

[1] Americans for Nonsmokers’ Rights Press Release: Celebrating 25 Years of Smokefree Skies. http://www.webcitation.org/6XrWQFILS.

[2] Holm A.L., Davis R.M. (2004). Clearing the airways: Advocacy and regulation for smoke-free airlines. Tob. Control.

[3] Lopipero P., Bero L.A. (2006). Tobacco interests or the public interest: 20 Years of industry strategies to undermine airline smoking restrictions. Tob. Control.

[4] Beatty A.L., Haight T.J., Redberg R.F. Associations between Respiratory Illnesses and Secondhand Smoke Exposure in Flight Attendants: A Cross-Sectional Analysis of the Flight Attendant Medical Research Institute Survey. http://www.ncbi.nlm.nih.gov/pubmed/21943016.

[5] Ebbert J.O., Croghan I.T., Schroeder D.R., Murawski J., Hurt R.D. Association between respiratory Tract Diseases and Secondhand Smoke Exposure among Never Smoking Flight Attendants: A Cross-Sectional Survey. http://www.ncbi.nlm.nih.gov/pmc/articles/PMC2064907/.

[6] Nagda N.L., Koontz M.D. (2003). Review of studies on flight attendant health and comfort in airliner cabins. Aviat. Space Environ. Med..

[7] Pinkerton L.E., Waters M.A., Hein M.J., Zivkovich Z., Schubauer-Berigan M.K., Grajewski B. (2012). Cause-specific mortality among a cohort of U.S. flight attendants. Am. J. Ind. Med..

[8] McNeely E., Gale S., Tager I., Kincl L., Bradley J., Coull B., Hecker S. The Self-Reported Health of U.S. Flight Attendants Compared to the General Population. http://www.ncbi.nlm.nih.gov/pmc/articles/PMC4007523/.

[9] Repace J. (2004). Flying the smoky skies: Secondhand smoke exposure of flight attendants. Tob. Control.

[10] Stillman F.A., Soong A., Kleb C., Grant A., Navas-Acien A. A Review of Smoking Policies in Airports around the World. http://www.ncbi.nlm.nih.gov/pubmed/24638966.

[11] Kungskulniti N., Charoenca N., Peesing J., Trangwatana S., Hamann S., Pitayarangsarit S., Chitanondh H. Assessment of Secondhand Smoke in International Airports in Thailand, 2013. http://www.ncbi.nlm.nih.gov/pubmed/24638967.

[12] Lee K., Hahn E.J., Robertson H.E., Whitten L., Jones L.K., Zahn B. (2010). Air quality in and around airport enclosed smoking rooms. Nicotine Tob. Res..

[13] Wagner J., Sullivan D.P., Faulkner D., Fisk W.J., Alevantis L.E., Dod R.L., Gundel L.A., Waldman J.M. (2004). Environmental tobacco smoke leakage from smoking rooms. J. Occup. Environ. Hyg..

[14] Centers for Disease Control and Prevention (CDC) (2012). Indoor air quality at nine large-hub airports with and without designated smoking areas—United States, October–November 2012. Morb. Mortal. Wkly. Rep..

[15] Pion M., Givel M.S. (2004). Airport smoking rooms don’t work. Tob. Control.

[16] Jones M., Wipfli H., Shahida S., Avila-Tang E., Samet J., Breysse P.N., Navas-Acien A. (2013). Secondhand Tobacco Smoke: An Occupational Hazard for Smoking and Non-Smoking Bar and Nightclub Employees. Tob. Control.

[17] Shargorodsky J., Zheng L., Stillman F., Soong A., Navas-Acien A., Reh D. Airline flight time and sinus disease; the secondhand-smoke, air quality and respiratory health among flight attendants survey. Int. Forum Allergy Rhinol..

[18] Localio A.R., Margolis D.J., Berlin J.A. (2007). Relative risks and confidence intervals were easily computed indirectly from multivariable logistic regression. J. Clin. Epidemiol..

[19] Americans for Nonsmokers’ Rights Secondhand Smoke: The Science. http://www.webcitation.org/6XxKCbzpA.

[20] Cho H., Lee K., Hwang Y., Richardson P., Bratset H., Teeters E., Record R., Riker C., Hahn E.J. (2014). Outdoor tobacco smoke exposure at the perimeter of a tobacco-free university. J. Air Waste Manag. Assoc..

[21] Klein E.G., Kennedy R.D., Berman M. (2014). Tobacco Control Policies in Outdoor Areas of High Volume American Transit Systems. J. Community Health.

[22] The Moscow Times: Smoking Ban Spreads to Moscow’s Sheremetyevo Airport. http://www.webcitation.org/6XxK71GGT.

[23] Legacy Tobacco Documents Library Young P: Patricia Young American Airlines Flight Attendant May 18, 1994. http://www.webcitation.org/6XxKFvA0C.

[24] Sirichotiratana N., Yogi S., Prutipinyo C. (2013). Perception of tourists regarding the smoke-free policy at suvarnabhumi international airport, Bangkok, Thailand. Int. J. Environ. Res. Public Health.

